# A systematic screen for genes expressed in definitive endoderm by Serial Analysis of Gene Expression (SAGE)

**DOI:** 10.1186/1471-213X-7-92

**Published:** 2007-08-02

**Authors:** Juan Hou, Anita M Charters, Sam C Lee, Yongjun Zhao, Mona K Wu, Steven JM Jones, Marco A Marra, Pamela A Hoodless

**Affiliations:** 1 Terry Fox Laboratory, British Columbia Cancer Agency, Vancouver, Canada; 2 Michael Smith Genome Sciences Centre, British Columbia Cancer Agency, Vancouver, Canada; 3 Department of Medical Genetics, University of British Columbia, Vancouver, Canada; 4 Terry Fox Laboratory, British Columbia Cancer Agency, 675 West 10^th ^Avenue, Vancouver, BC, V5Z 1L3, Canada

## Abstract

**Background:**

The embryonic definitive endoderm (DE) gives rise to organs of the gastrointestinal and respiratory tract including the liver, pancreas and epithelia of the lung and colon. Understanding how DE progenitor cells generate these tissues is critical to understanding the cause of visceral organ disorders and cancers, and will ultimately lead to novel therapies including tissue and organ regeneration. However, investigation into the molecular mechanisms of DE differentiation has been hindered by the lack of early DE-specific markers.

**Results:**

We describe the identification of novel as well as known genes that are expressed in DE using Serial Analysis of Gene Expression (SAGE). We generated and analyzed three longSAGE libraries from early DE of murine embryos: early whole definitive endoderm (0–6 somite stage), foregut (8–12 somite stage), and hindgut (8–12 somite stage). A list of candidate genes enriched for expression in endoderm was compiled through comparisons within these three endoderm libraries and against 133 mouse longSAGE libraries generated by the Mouse Atlas of Gene Expression Project encompassing multiple embryonic tissues and stages. Using whole mount *in situ *hybridization, we confirmed that 22/32 (69%) genes showed previously uncharacterized expression in the DE. Importantly, two genes identified, *Pyy *and *5730521E12Rik*, showed exclusive DE expression at early stages of endoderm patterning.

**Conclusion:**

The high efficiency of this endoderm screen indicates that our approach can be successfully used to analyze and validate the vast amount of data obtained by the Mouse Atlas of Gene Expression Project. Importantly, these novel early endoderm-expressing genes will be valuable for further investigation into the molecular mechanisms that regulate endoderm development.

## Background

The definitive endoderm (DE) is a population of multi-potent stem cells allocated as one of the primary germ layers during gastrulation. Initially formed as an epithelial sheet of approximately 500–1000 cells around the distal cup of an E7.5 mouse embryo, the DE is rapidly organized into a tube that runs along the anterior-posterior axis of the embryo [1-3]. The DE gives rise to the major cell types of many internal organs, including the thyroid, thymus, lung, stomach, liver, pancreas, intestine and bladder. Most of these organs have secretory and/or absorptive functions and play important roles in controlling body metabolism. Interest in the endoderm has intensified recently because processes that govern early development of DE-derived tissues may be recapitulated during stem cell differentiation [4,5], which could provide future therapies for diseased adult organs. Understanding how DE-derived organs are specified, differentiate, proliferate, and undergo morphogenesis is key to understanding visceral organ disorders and tissue regeneration.

The last decade has yielded great insights into the molecular regulation of DE development [6]. In particular, pathways governing the initial formation of DE, patterning of the foregut, and morphogenesis of foregut-derived organs such as the pancreas and liver, have begun to be deciphered. Many of the key genes involved in the initial formation of DE are evolutionarily conserved. They include Nodal and components of its signaling pathway, transcription factors of the mix-like paired homeodomain class, Forkhead domain factors, and Sox17 HMG domain proteins [7-11]. Studies of ventral foregut patterning suggest that endoderm patterning is controlled by soluble factors provided by an adjacent germ layer [12]. FGF4, which is expressed in the neighboring cardiac mesoderm, can induce the differentiation of ventral foregut endoderm in a concentration-dependent manner [13,14]. FGF2 and Activin, secreted by the notochord, lead to the expression of pancreatic markers by repressing expression of *Shh *in pancreatic endoderm [15-19]. However, the precise hierarchical relationships between these factors and their downstream targets are still largely unknown, and complete molecular hierarchies have not been obtained. In addition, midgut and hindgut development is largely unexplored.

Embryonic stem (ES) cells have attracted much attention as a possible source of cells for regenerative medicine. Directing differentiation efficiently into specific lineages at high purities from ES cells requires both optimal selective culture conditions and markers to guide and monitor the differentiation process. While several methods of differentiation of ES cells to hepatic and insulin-producing cells have been described, determining the precise identity of these cells is problematic due to a lack of suitable markers [20-23]. More recently, two groups achieved efficient differentiation of human and murine ES cells into DE by combining directive culture conditions (serum concentration reduction and Activin supplements) and FACS sorting using the cell surface marker, CXCR4 [4,5,24]. Although useful, CXCR4 is not an ideal marker for the DE as it is widely expressed in the gastulation stage mouse embryo (Table 1 and [5,25]). At present there is no DE-specific marker that can unequivocally identify this cell type.

**Table 1:** Tag counts for endoderm and ectoderm genes in the endoderm and ectoderm SAGE libraries.

**gene symbol**	**tag sequence**	**Early Endoderm **(108579)	**Foregut **(102972)	**Hindgut **(110529)	**Neural tube **(97364)	**Anterior neuropore **(103594)	**Posterior neuropore **(102196)
**endoderm genes**							

*Cxcr4*	TGAATGAGTGTCTAGGC	5	1	6	2	2	2
*Ecad*	TAATGTTGCTAGAGTGA	9	9	8	0	0	1
*Foxa1*	TTAACGACAAAAAAAAA	5	4	1	0	1	0
*Foxa2*	GTGAAATCCAGGTCTCG	8	6	9	1	2	0
*Foxa3*	CTGCTATGCACCAAGAT	2	1	3	0	0	0
*Gata4*	CCTGCCCCTCCTCCACA	1	2	1	0	0	0
*Gata6*	TACACAATAATTTTTTT	3	6	3	0	0	0
*Hhex*	TATATAGCATTACTTCT	2	4	1	0	0	0
*Ihh*	GGAGAATTTTGGGAATG	2	0	4	0	0	0
*Shh*	TTCTTGGAAACCAAGAC	11	10	7	1	0	0
*Sox17*	CGTGTTTTCTCAATCTT	21	2	8	0	0	0

**ectoderm genes**							

*Fgf15*	ACTTGTTTTCTACATTA	0	0	0	1	4	2
*Hes5*	TGGGAGAACACAGGCTG	0	0	0	1	3	2
*Ncad*	TTAATATCTTTCGTTAT	0	0	1	3	2	3
*Pax6*	GATTTAAGAGTTTTATC	0	0	0	4	1	1
*Sox2*	TATATATTTGAACTAAT	1	3	1	6	5	10
*Sox3*	TACCTGCCACCTGGCGG	0	0	0	3	4	3
*Zic2*	TGATGTTTCAGTGCTTT	0	0	0	6	4	4
*Zic3*	AATAACAGAAAAGTGGA	0	0	0	1	1	0

The total number of tags in each library is bracketed in the column heading.

In summary, one major hurdle in the analysis of early DE development in both the embryo and ES cells is the lack of both pan-endodermal and endodermal region-specific genetic markers, since the majority of DE markers are also expressed in the visceral endoderm and/or other germ layers. Devising screens to identify genes specifically expressed in DE will contribute to studies of DE development. Several groups have carried out screens for novel genes expressed in the endoderm of *Xenopus *and mouse embryos using microarray or cDNA hybridization [25-29]. Despite the identification of several endoderm enriched genes, no novel DE specific genes were identified. As an alternative approach, we used Serial Analysis of Gene Expression (SAGE) to provide quantitative gene expression profiles. SAGE has been improved by the development of a longSAGE protocol, which generates tags that are 21 bp long and provides enhanced efficiency and accuracy of tag-to-gene mapping [30-32]. Compared with microarrays, SAGE has the additional advantage that it permits the identification of novel transcripts. SAGE also has the added benefit that the data are digital and thus can be easily shared among investigators and compared across different experiments and tissues.

In this study, we generated and analyzed three mouse DE longSAGE libraries. A list of candidate genes enriched for expression in endoderm was compiled through comparisons within these three endoderm libraries and against 133 mouse longSAGE libraries representing multiple embryonic stages and tissues generated by the Mouse Atlas of Gene Expression Project [32,33]. Sixty nine percent of these candidate genes showed previously uncharacterized expression in restricted tissues, including DE, after further whole mount *in situ *hybridization validation. Importantly, two genes identified, *Pyy *and *5730521E12Rik*, showed exclusive DE expression at early stages of endoderm patterning. The high efficiency of this screen suggests that our endoderm libraries and the SAGE library database are powerful resources to identify tissue specific genes. Furthermore, these new endoderm genes provide a valuable tool for further investigation into the molecular mechanisms regulating endoderm development.

## Results

### Overview of the endoderm libraries

**Figure 1:**
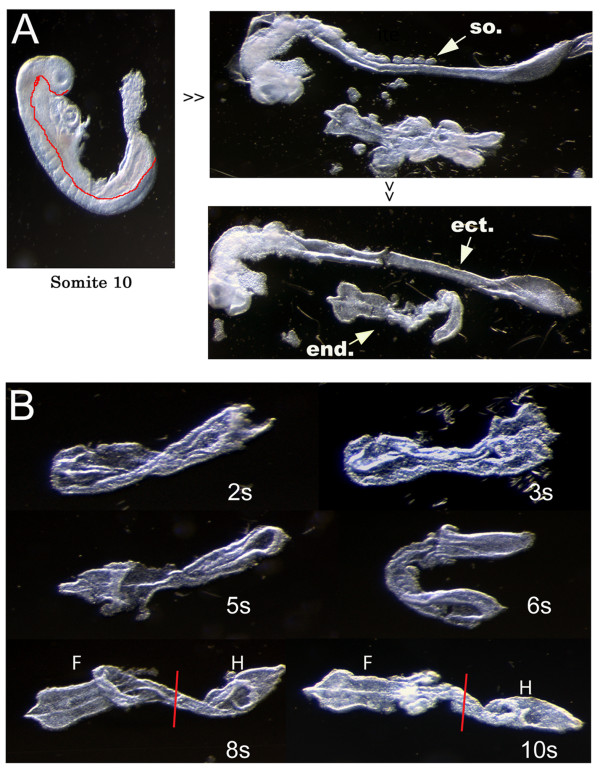
Collection of definitive endoderm from E8.0–8.5 mouse embryo. (A) Dissection procedure and germ layer separation process. After trypsin treatment, ectoderm and endoderm can be separated (indicated by the red line). After the somites and mesoderm were removed, enriched ectoderm and endoderm can be obtained. (B) Photographs of the intact dissected endoderm at the indicated somite stage. Somite 0–6 endoderm pieces were pooled for the early whole endoderm library. Somite 8–12 endodermal portions were separated into foregut and hindgut portions (indicated by the red line). So: somite; end: endoderm; ect: ectoderm; F: foregut; H: hindgut.

Enriched definitive endoderm tissue was obtained by a combination of proteolysis and manual micro-dissection methods [14]. After removing the extra-embryonic region and digestion with trypsin, the DE was separated from ectoderm and mesoderm (Figure 1A). Somite 0–6 endoderm pieces were pooled for the early whole endoderm library (SM108, Figure 1A). At this stage the newly formed endoderm has not yet been patterned, based on endoderm explant experiments [14,34]. Somite 8–12 endoderm was divided through the midgut into foregut and hindgut regions, and then pooled for the foregut and hindgut libraries respectively (SM107 and SM112, Figure 1B). By this stage endoderm patterning has initiated [14,35]. The notochordal plate at 0–6 somite stage and the notochord at 8–12 somite stage adjoin the DE and thus were included in the library [36].

**Figure 2:**
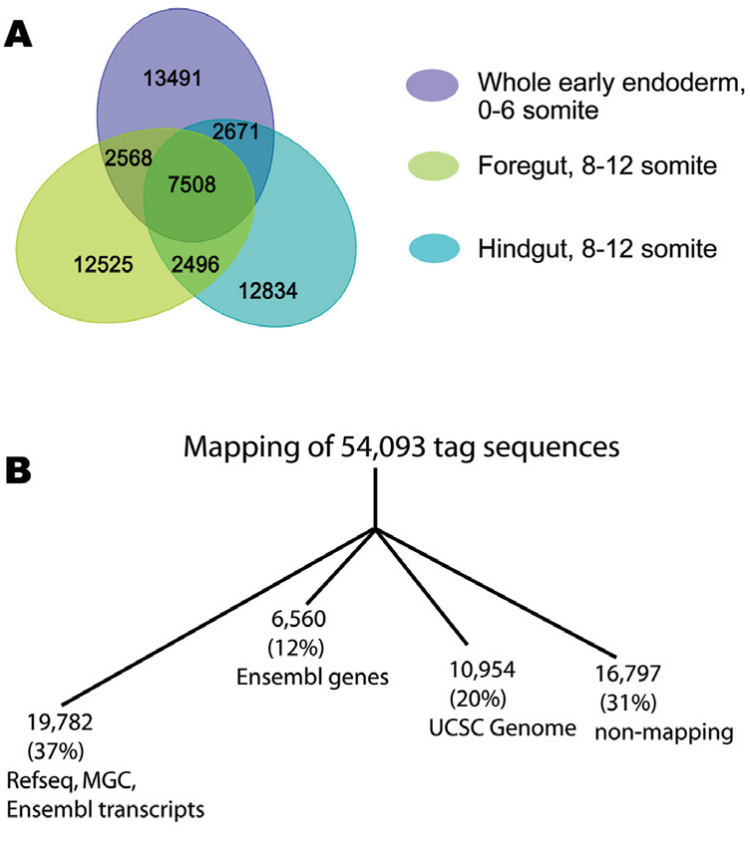
Overview of the endoderm SAGE libraries. (A) Venn diagram summarizing the number of unique and common tag-sequences in the three endoderm longSAGE libraries. (B) Summary of tag-to-gene mapping efficiencies. Additional details are in text.

A total of 322,208 tags were sequenced from these three longSAGE endoderm libraries [33]. Analysis of the three libraries revealed the expression of 54,093 different tag-sequences (see Methods). There were 26,238 tag-sequences present in the early endoderm library (SM108). Of these tag-sequences, 51% were unique to the early endoderm library as compared to the later endoderm libraries. Similarly, 25,097 and 25,509 tag-sequences were present in the foregut (SM107) and hindgut (SM112) libraries, respectively. In each of these libraries, approximately 50% of the tag-sequences were unique, compared to the other two endoderm libraries (Figure 2A). To determine which genes the tag-sequences represented, we first compared our tag-sequences to transcript databases (Refseq, MGC and Ensembl). Tag-sequences that did not correspond to annotated transcripts were then mapped to Ensembl gene units, which were extracted from the Ensembl database and include intronic regions and 1.0 kb upstream and downstream of annotated transcripts (Ensembl genes). Finally, tags were mapped to the mouse genome (UCSC). Of the combined 54,093 tag-sequences, 37% (19,782) mapped to known transcripts using the Refseq, MGC and Ensembl transcript databases, 12% (6,560) mapped to known genes using the Ensembl genes, implicating alternative splicing and alternative 3' UTRs of known genes, and 20% (10,954) mapped to the mouse genome. The remaining 31% (16,797) of the tag-sequences did not map to any of these databases (Figure 2B). Ninety percent of these unmapped tag-sequences were single tags, implying that many may have been generated by sequencing, PCR, or other errors. We have previously shown that many of these tag-sequences can be mapped by allowing a one-basepair mismatch, insertion or deletion [33]. However, some of these tag-sequences likely represent valid, novel transcripts, since 44 unmapped tag-sequences expressed in the endoderm were found at a level of at least 4 tags. For example, these 44 tag-sequences may span an unknown splice junction [37]. To simplify the analysis and validation in this study, we focused on tag-sequences that unambiguously mapped to the most 3' position (position number +1) and the sense strand of the Refseq database (refer to Methods); 7,084 tag-sequences (13%) met these criteria.

To assess the quality of our endoderm libraries, we searched for genes known to be expressed in the endoderm (*Cxcr4, Ecad*, *Foxa1-3, Gata4*, *Gata6, Hhex, Ihh, Shh*, and *Sox17*) and ectoderm (*Fgf15, Hes5, Ncad, Pax6, Sox2, Sox3, Zic2, Zic3*) (Table 1). Since we also generated 3 ectoderm libraries from early somite stage mouse embryos, we evaluated the integrity of the libraries by comparing gene expression levels in the endoderm and ectoderm libraries. Significantly, all of these endoderm genes were present in our endoderm libraries and excluded or present at low levels in the ectoderm libraries. The exception is *Cxcr4*, which although used as a DE marker, was expressed in both endoderm and ectoderm, reaffirming it as widely expressed [25]. Similarly, *Sox2 *is expressed in both ectoderm and endoderm libraries corresponding to published expression patterns [38]. All of the other ectoderm genes present in our ectoderm libraries were excluded or present at low level in the endoderm libraries. Overall, the expression patterns observed in our libraries supports known expression data for these genes, indicating that the libraries are representative of endoderm and ectoderm transcription.

### Identification of foregut-specific genes

**Figure 3:**
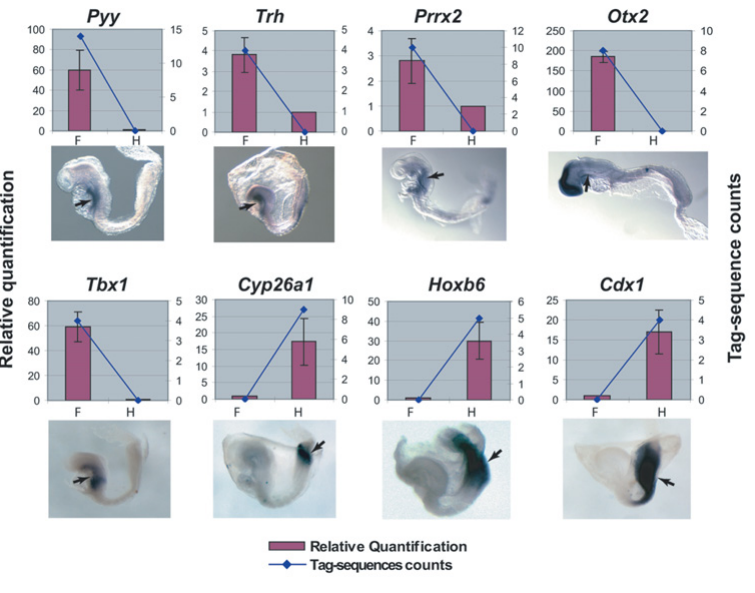
Correlation of the expression validation of 8 genes from the first list between RT-qPCR, whole mount *in situ *hybridization and SAGE. For each gene, the upper panel shows the comparison of expression level using RT-qPCR and SAGE (Left scale: relative quantification indicated by the bars; Right scale: raw tag-sequence counts indicated by the line. F: foregut; H: hindgut). The lower panel shows the expression pattern detected by whole mount *in situ *hybridization. For all embryos, anterior is to the left and posterior is to the right. The RT-qPCR, whole mount *in situ *hybridization and SAGE validation results were well correlated. *pYY*, *Trh*, *Prrx2*, *Otx2 *and *Tbx1 *are highly expressed in the foregut (indicated by arrow). Conversely, *Cyp26a1*, *Hoxb6 *and *Cdx1 *are highly expressed in the hindgut (indicated by arrow).

To identify genes that were specifically expressed in the foregut or the hindgut, a cross-comparison between the two libraries (SM107 and SM112, respectively) was performed. An initial list of genes was made by selecting tag-sequences that were present at counts ≥4 for transcription factors (TFs) and signaling pathway components (SPCs), and counts ≥7 for other genes, in either the foregut or hindgut library. This threshold allowed us to identify the top 25 most highly expressed tag-sequences present exclusively in the foregut library and the top 20 most highly expressed tag-sequences present exclusively in the hindgut library, which was a tractable number for further validation [see Additional file 1]. By screening with both semi-quantitative RT-PCR and quantitative RT-PCR, 14 of the 45 genes were shown to exhibit differential expression between the foregut and hindgut. Whole mount *in situ *hybridization was performed on these 14 genes. Six of these genes showed a ubiquitous expression pattern, making it difficult to determine whether there was differential expression within the DE. However, 8 genes did exhibit differential expression levels between the foregut and hindgut (Figure 3). Seven of these genes, *Trh*, *Otx2*, *Prrx2*, *Tbx1*, *Cyp26a1*, *Hoxb6*, and *Cdx1 *were expressed in other tissues as well as endoderm at the early somite stage. Significantly, one of the genes, *Pyy*, was exclusively expressed in the foregut endoderm.

### Expression of *Pyy *in the early mouse embryo

**Figure 4:**
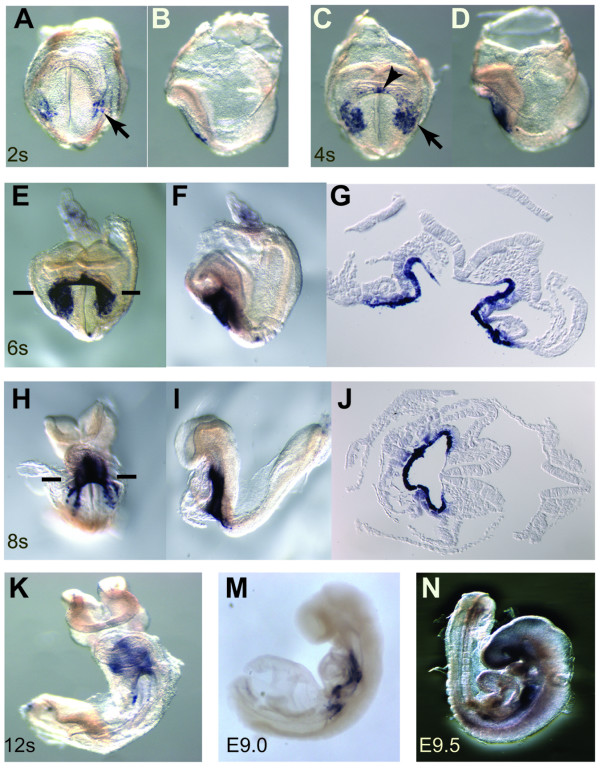
Expression of *Pyy *in the early developing mouse embryo. (A, B) *Pyy *expression is seen in small lateral region of the DE at as early as 2 somite stage (indicated by arrow). (C, D) At the 4 somite stage, the expression domains in the lateral region are expanded, and the second expression domain which is in the medial ventral foregut can to be observed (arrowhead). (E-J) The lateral expression domains expanded and extended anteriorly to the medial ventral foregut. Strong expression was observed in the lateral and ventral foregut in the 6–8 somite stages. Representative sections are shown in the right panel. (K-N) In the early organogenesis stage, the *Pyy *expression remained in the posterior foregut extending to the midgut junction.

*Pyy *is known to be highly expressed in pancreatic islets and endocrine L cells of the lower gastrointestinal tract [39], but its early embryonic expression pattern has not been described. Due to the exclusive expression of *Pyy *in the DE at early somite stages from our analysis, we further examined *Pyy *expression pattern during early mouse embryogenesis. Whole mount *in situ *hybridization was performed on embryos collected from E6.0 to E9.5 stages (Figure 4). Interestingly, *Pyy *was expressed in small lateral regions of the foregut DE as early as the 2 somite stage (Figure 4A, 4B). At the 4 somite stage, the expression domains in the lateral region were expanded and a second expression domain in the medial ventral foregut was observed (Figure 4C, 4D). Subsequently, the lateral expression domains expanded and extended anteriorly to the medial ventral foregut, so that strong expression was observed in the lateral and ventral foregut at the 6–8 somite stages (Figure 4E–J). Interestingly, the expression was restricted to the posterior half of the foregut and never observed in the anterior half of the foregut pocket. At early organogenesis stage, *Pyy *expression remained in the posterior foregut extending to the midgut junction (Figure 4K–N). Thus, *Pyy *is expressed earlier than previously reported and demonstrates a dynamic expression pattern in the early DE.

### Identification of novel genes expressed in the DE

**Figure 5:**
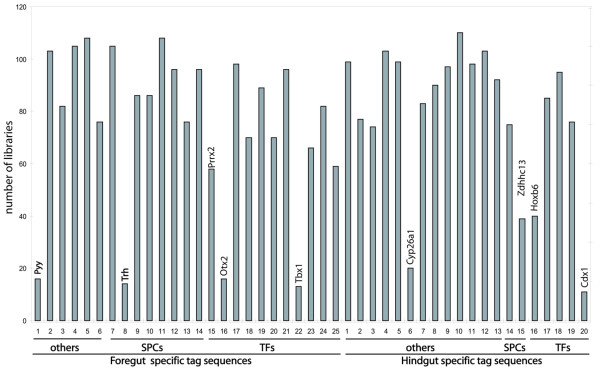
Expression of genes from the first candidate list within 133 mouse atlas SAGE libraries. Numbers on the X-axis depict each tag-sequence in the first candidate list, and the Y-axis depicts the number of the libraries in which a specific tag-sequence is present. Thus a low bar reflects high tissue specificity, and *vice versa*. The 8 genes from the 1^st ^list exhibiting differential expression between foregut and hindgut by RT-qPCR as well as whole mount *in situ *hybridization have significantly lower bars. Names of these genes are provided on top of their respective bars. SPCs: Signaling Pathway Components; TFs: Transcription Factors.

In addition to identifying foregut- and hindgut-enriched DE markers, we wanted to identify additional novel genes with distinct expression patterns in the endoderm to facilitate DE patterning studies. Thus, to increase the efficiency of identification of novel endoderm genes, we chose to exploit the Mouse Atlas of Gene Expression Project database, which contained 133 libraries from different tissues and stages of development. We reasoned that if a gene was ubiquitously expressed, it would be present in most of the libraries. Conversely, if the expression of a gene were restricted to a specific cell-type, it would be present only in a specific subset of libraries. Indeed, by examining the expression patterns of our original list (foregut vs hindgut) of 45 tag-sequences in 133 longSAGE libraries generated by the Mouse Atlas Project, we discovered 9 genes that exhibited high tissue-specificity since they were present in only a few libraries (Figure 5). Interestingly, 8 of the 9 genes demonstrating a tissue-restricted expression pattern matched the endoderm genes identified in our *in situ *hybridization analysis (Figure 3). This suggests that in the context of looking for specificity of gene expression, the SAGE data is an excellent tool for identifying genes with tissue restricted expression.

To identify genes expressed in the DE, a second list was generated using tag-sequences present in the three endoderm libraries (7,084 tag sequences which were unambiguously mapped to the most 3' position and the sense strand of the Refseq database). We considered two factors, the total number of Mouse Atlas SAGE libraries in which a tag-sequence was present (L), and the total number of times that a tag-sequence was found in the three pooled endoderm libraries (T). We rationalized that higher T values and lower L values and thus higher T/L ratio would correspond to the degree of the endoderm-enrichment. We compiled a list consisting of tag-sequences with T>4 and L<58 and calculated the T/L ratio [see Additional file 2]. We removed the tag sequences whose T/L ratio was less than 0.21 to create a second list consisting of 60 genes. Confirming the effectiveness of these criteria, 6 out of the 60 genes were present in and validated by our first list, and 24 out of the 60 genes were previously shown to be expressed in endoderm, either with or without expression in other germ layer tissues, including *Sox17*, *Foxa1-3*, *Ihh *and *Shh *[see Additional file 2].

**Figure 6:**
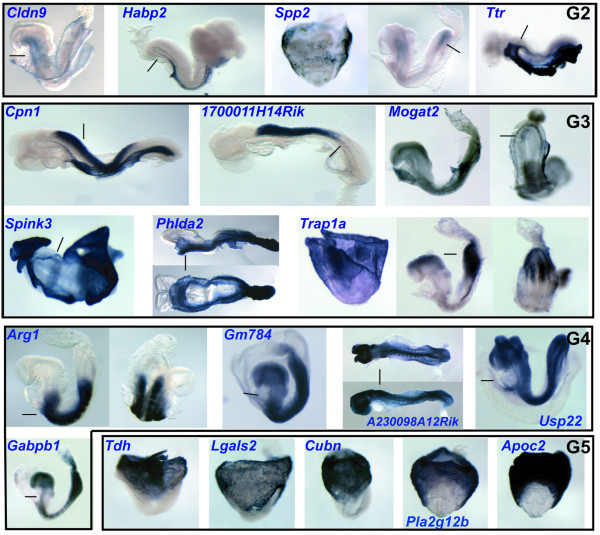
Whole mount *in situ *hybridization validation of the 2^nd ^candidate list, illustrating the complex expression patterns of endoderm genes. For explanation see text and Table 2. The DE expressions of the genes in Groups 2–4 are shown further by histological sections in Figure 7.

**Figure 7:**
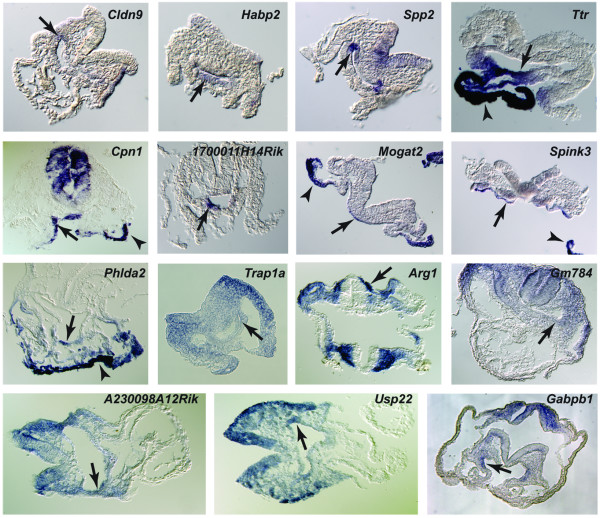
Histological sections through the embryos as indicated by the line in Figure 6, with arrows pointing to the DE staining. Arrowheads indicate staining in visceral endoderm.

Of the remaining 30 genes, we successfully examined the expression of 26 genes using whole mount *in situ *hybridization. 21 of the 26 genes showed tissue-restricted expression patterns (Figure 6, 7 and Table 2), while the remaining 5 genes showed ubiquitous expression at E8.5. Including the 6 candidate genes validated from the first list, the efficiency of our new screen for novel genes with tissue-restricted expression patterns was 84% (27/32). Interestingly, we found the majority of genes identified were not only expressed in the definitive endoderm, but also in other tissues such as yolk sac, ectoderm and mesoderm. We classified the 27 genes exhibiting tissue-restricted expression into five categories, based on their expression patterns (Table 2). The first group includes two genes, *5730521E12Rik *and *Pyy*, which were expressed exclusively in the DE. We described the *Pyy *expression pattern in the early mouse embryo above and, the *5730521E12Rik *expression pattern is described below. Group 2 included genes that were expressed in the definitive endoderm and yolk sac endoderm, which support the functional similarity between these two lineages [6]. Group 3 contained genes that were expressed in the DE, yolk sac and another germ layer with a tissue-restricted pattern, and Group 4 contained genes that were not expressed in yolk sac and heart, but expressed in all 3 germ layers. The genes in Group 5 were expressed in yolk sac endoderm at high levels, without obvious expression within the DE. The tags for these genes may be included in our libraries due to yolk sac endoderm contamination, which is difficult to avoid when collecting the DE tissue. Alternatively, these genes may be expressed in the DE at low levels but their expression in DE could be under-estimated by *in situ *hybridization due to very high levels in yolk sac endoderm. Thus most of the genes selected by our criteria for *in situ *hybridization showed complex tissue-restricted expression patterns in the early embryo, including the DE. Overall, these results indicate our approach was successful in the identification of novel markers of endoderm expression.

**Table 2:** Tissue restricted expression of the genes isolated by whole mount *in situ *hybridization.

**Gene symbol**	**Gene name**	**L**	**F**	**E**	**H**	**T**	**Expression pattern in E8.0–8.5**
**Group1: Genes expressed in definitive endoderm only**							

*Pyy	peptide YY	19	14	9	0	23	foregut
*5730521E12Rik*	5730521E12Rik	9	9	25	63	97	midgut

**Group2: Genes expressed in both definitive and yolk sac endoderm**							

*Cldn9*	Claudin9	18	4	4	1	9	definitive endoderm and yolk sac
*Habp2*	Hyaluronic binding protein 2	25	0	2	4	6	definitive endoderm and yolk sac
*Spp2*	Secreted phosphoprotein2	16	1	7	2	10	definitive endoderm and yolk sac
*Ttr*	Transthyretin	29	4	1	7	12	definitive endoderm and yolk sac

**Group3: Genes expressed in definitive endoderm, yolk sac and other germ layers**							

*Cpn1*	Carboxypeptidase N, polypeptide1	28	2	5	6	13	definitive endoderm, neural tube and yolk sac
*1700011H14Rik*	1700011H14Rik	27	1	2	4	7	definitive endoderm(weak), neural tube and yolk sac
*Mogat2*	Monoacylglycerol O-acyltransferase2	36	2	11	6	19	yolk sac endoderm, definitive endoderm, anterior ectoderm, and somite
*Spink3*	Serine peptidase inhibitor, Kazal type3	38	44	109	57	210	yolk sac endoderm, ectoderm and definitive endoderm
*Phlda2*	Pleckstrin homology-like domain, family A, member2	26	7	10	5	22	yolk sac endoderm, lateral plate mesoderm and ventral definitive endoderm
*Trap1a*	Tumor rejection antigen P1A	17	1	5	6	12	definitive endoderm, yolk sac and midbrain and tailbud

**Group4: Genes not expressed in yolk sac and heart, but expressed in other germ layers**							

*Gabpb1*	GA repeat binding protein, beta1	27	3	1	2	6	entire definitive endoderm, head and tailbud ectoderm and mesoderm, but not expressed at heart and yolk sac
*Cdx1	Caudal type homeo box1	13	0	17	4	21	3 germ layers of the posterior embryo
*Trh	Thyrotropin releasing hormone	18	4	9	0	13	definitive endoderm, brain, midline
*Cyp26a1	Cytochrome P450, family 26, subfamily a, polypeptide1	24	0	5	9	14	tailbud
*Tbx1	T-box1	14	4	1	0	5	foregut and mesoderm
*Otx2	Orthodenticle homolog2	27	8	2	0	10	brain, foregut
*Arg1*	Arginase 1, liver	15	4	0	4	8	3 germ layers of the trunk, but not expressed at head, heart, tail bud and yolk sac
*Gm784*	Gene model 784	37	6	1	5	12	3 germ layers, but not expressed at heart and yolk sac
*A230098A12Rik*	A230098A12Rik	10	3	1	1	5	3 germ layers, but not expressed at heart and yolk sac
*Usp22*	Ubiquitin specific peptidase	38	2	3	3	8	3 germ layers, but not expressed at heart and yolk sac

**Group5: Genes expressed in yolk sac endoderm only**							

*Tdh*	L-threonine dehydrogenase	19	1	7	11	19	yolk sac endoderm
*Lgals2*	Lectin, galactose-binding, soluble 2	18	1	2	6	9	yolk sac endoderm
*Cubn*	Cubilin (intrisic factor-cobalamin receptor)	23	1	16	17	23	yolk sac endoderm
*Pla2g12b*	Phospholipase A2, group12B	16	2	2	1	5	yolk sac endoderm
*Apoc2*	ApolipoproteinC-2	17	3	13	7	23	yolk sac endoderm

L: number of libraries which the 3' most tag sequence of each gene present in; F, E, H, T: raw counts of the 3' most tag sequences for each gene in foregut library (F), whole endoderm library (E), hindgut library (H) and in the three endoderm libraries (T) respectively. *: genes validated in the first list.

### Expression of *5730521E12Rik *in early mouse embryo

**Figure 8:**
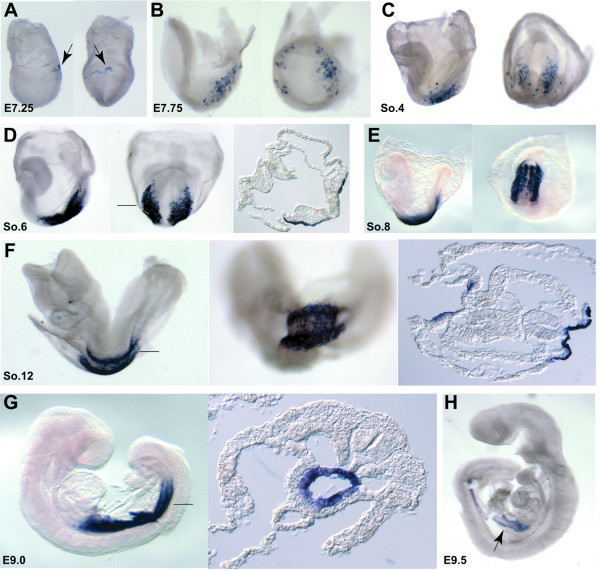
Expression of *5730521E12Rik *in the early developing mouse embryo. *5730521E12Rik *expression from E6.5 to E9.5 in the mouse embryo was examined by whole mount *in situ *hybridization. The embryos at each represented stage are shown in lateral and posterior view (A-D) or lateral and ventral view (E, F), except E9 and E9.5 (G, H). For the lateral view, the embryos are oriented so that the anterior is to left. The expression of *5730521E12Rik *is dynamic. Hybridization signals initiate at E7.25 in a few cells in the posterior of the embryos (arrowheads in A), then is continuously detected in broader bilateral domains of the middle-posterior of the embryos at head fold (B) and early somite (C-E) stages. At E8.5–E9 (E-G), *5730521E12Rik *expression level reaches the highest in the midgut. At E9.5, the signal is retained but is down-regulated (H).

In addition to *Pyy*, *5730521E12Rik *exhibited exclusive expression in the DE at early somite stages in our analysis. We further examined the expression pattern of *5730521E12Rik *during early mouse embryogenesis. Interestingly, *5730521E12Rik *was first expressed in a few cells at E7.25 in the endoderm at the posterior region adjacent to the embryonic-extraembryonic junction (Figure 8A). At the late head-fold stage, *5730521E12Rik *expression has expanded in the lateral region of the endoderm on the posterior side (Figure 8B). As development proceeds, the bilateral expression domains extended anteriorly and medially and began to focus in the midgut region at early somite stages (Figure 8C–F). By E9.0 strong expression was observed in the midgut (Figure 8G). At E9.5, *5730521E12Rik *expression was still maintained in the midgut region with the expression level decreased (Figure 8H). Thus *5730521E12Rik *expression was specific to the midgut during the gastrulation and early organogenesis stages in the mouse embryo.

## Discussion

Formation, specification and patterning of the definitive endoderm are poorly understood in the embryo compared to other germ layers. Due to a lack of exploratory tools to aid these studies, interest in the identification of novel endoderm genes is growing. The recent enthusiasm for stem cell differentiation methodologies and the clinical potential for these cells have heightened the need for better tools and a further understanding of normal embryonic development. Several groups have undertaken large-scale screens to identify novel genes that may be informative for developmental processes. In particular, *in situ *hybridization has been used to identify novel genes with unique expression patterns at mid-gestation (E9.5) [29]. However, while *in situ *hybridization is considered to be the ultimate and proven method to validate tissue-specific genes, obtaining embryos for *in situ *hybridization at appropriate stages is more costly and time-consuming in mouse than in chicken, frog or fish. Thus ensuring high efficiency in the screening for tissue-specific genes during mouse development is an important consideration.

To identify novel definitive endoderm specific genes, we used a longSAGE approach. We were able to enhance our screening efficiency since the endoderm longSAGE libraries were generated from enriched DE tissues at early stages of DE formation (E8.0–E8.5) that were obtained by a combination of proteolytic and manual micro-dissection methods. In addition, pre-selection of candidate genes by comparisons with 133 SAGE libraries from various tissues allowed us to eliminate ~95% of the widely expressing genes from our endoderm libraries. Overall, the efficiency of our screen for genes with DE expression was 69% (22/32, not including Group 5 genes which are highly expressed in the yolk sac). By including genes expressed in the yolk sac visceral endoderm, we observed 84% (27/32) of genes identified with endoderm expression. Significantly, two of these genes, *Pyy *and the Riken gene, *5730521E12Rik*, were exclusively expressed in the DE lineage at early organogenesis stages of development.

Previous studies focusing on screening for novel endoderm genes have used cDNA cloning or microarray analysis [25-29,40]. Sousa-Nunes *et al*. identified 29/160 (18%) genes with restricted expression patterns from E7.5 mouse endoderm cDNA libraries using non-redundant sequence-based selection and *in situ *hybridization, but not all of these genes were endoderm enriched in their expression [40,41]. Sherwood *et al*. recently used fluorescent activated cell sorting to isolate definitive and visceral endoderm cell populations for microarray analysis [25]. They identified 18 out of 27 (67%) novel genes whose expression was enriched in endoderm. They defined a pan-endodermal signature composed of 22 novel and known genes that is preferentially expressed in definitive and visceral endoderm. Interestingly, neither study was able to identify novel genes that are expressed specifically in the DE.

The lack of DE specific genes may be due to sensitivity and depth of screening. Furthermore, the high functional similarity between visceral and definitive endoderm suggests that these tissues have highly related transcriptomes [25]. Several genes in our study were found to be expressed in both visceral and definitive endoderm, supporting the similarity of the two tissues. It is likely that some endoderm-specific or enriched genes were excluded from the gene list determined by our selection criteria. Our SAGE sampling depth (~100,000 tags per library) yields gene-detection sensitivity approximately equivalent to that of fluorescence-based microarray approaches [42], and is thus sufficient for detection of abundant and moderately abundant transcripts but is likely insufficient for reliable detection of rare transcripts. Several previously known foregut or hindgut markers were not present in our list likely due to their low expression level and/or their expression being restricted to few cells within the endoderm. For example, *Prox1 *is only expressed by the liver and pancreas progenitors beginning at the 7–8 somite stage [43]. Therefore, the *Prox1 *transcripts could be diluted by the total number of transcripts present in the foregut endoderm tissues, and thus not detected at our sequencing depth. With the advent of less expensive "next generation" sequencing this short-coming can be overcome by sequencing SAGE libraries to a greater depth. Furthermore, the foregut marker, *Hhex*, was missed in our analysis since it was expressed in 67 of our SAGE libraries, and thus did not fit our criteria for our validation lists (Table 1 and Additional file 2) [44]. Since many developmentally important genes are transcribed repeatedly and presumably function during multiple developmental processes, further refining of library and tissue choices for comparisons would be required to identify genes that are expressed in many stages of development and many tissues.

*Pyy *and *5730521E12Rik *were identified to be exclusively expressed in the DE lineage at gastrulation and early organogenesis stages of development. Initially, *Pyy *is expressed at the early somite stage in the bilateral and medial regions of the foregut. Its early regionalized expression within DE reflects an early specification of cell fate along both the anterior-posterior and lateral-medial axis of the embryonic gut [6,45]. Subsequently, *Pyy *is expressed in the posterior foregut extending to the midgut junction, and at later stages (E14.5-adult) expression becomes restricted to the pancreas, stomach and intestine (data not shown). Interestingly, Tremblay *et al*. recently tracked progenitor domains in the anterior endoderm of mouse embryos, using vital dyes to label those cells at 1–10 somite stage. They identified two distinct types of DE progenitor cells, lateral and medial, arising from three spatially separated embryonic domains. These domains converge to generate the epithelial cells of the liver bud [13]. Intriguingly, the expression of *Pyy *follows a similar pattern as that observed by the lineage tracing of the liver bud progenitors. However, pancreatic progenitors were rarely labeled by this lineage tracing [13] suggesting that *Pyy *may not mark the identical domain. Deletion of *Pyy *in mice does not reveal any obvious defects in endoderm patterning [46,47]. However, genetic lineage tracing using *Pyy-Cre *and a *ROSA26 *reporter mouse strain demonstrated that in the adult, descendants of *Pyy*-expressing cells can contribute to the periphery of pancreatic islets and the L-type cells of the distal intestine [46]. The relationship between these later descendants and the early expression patterns has not been explored. Regardless, the dynamic expression pattern of *Pyy *appears to reiterate the morphogenetic movement of foregut progenitors along anterior-posterior and medial-lateral axes prior to tissue specification.

The RIKEN gene, *5730521E12Rik*, expressed in the mid-gut region, is the first known gene that marks exclusively the entire midgut region at early organogenesis stages. Furthermore, *5730521E12Rik *is the earliest DE specific and regional marker reported to date. Its early regionalized expression in the few cells in the posterior DE at as early as E7.25 embryo may reflect the early specification of the DE. Tam *et al*. recently depicted the sequential allocation and global pattern of movement of the DE in the mouse embryo during gastrulation, by tracing cells electroporated with *Gfp *or painted with carbocyanine dyes [45]. The observations from their study, together with previous fate mapping studies, suggested a probable sequence of allocation of the DE proceeding with (a) the most-posterior endoderm and the dorsal endoderm of the rostral segment of the foregut at early-streak stage; (b) the ventral endoderm of the rostral foregut and additional posterior endoderm at the mid-streak stage; (c) the dorsal and then the ventral endoderm of the posterior segment of the foregut at the late-streak to late-bud stage; and finally, (d) the endoderm of the embryonic mid- and hind-gut at the late-bud to early head-fold stage [45,48-51]. Fascinatingly, the dynamic expression pattern of *5730521E12Rik *suggests that it may mark the last population of the DE precursors recruited, thus is possibly a midgut lineage marker. Interestingly, *5730521E12Rik *is identical to *nephrocan *(*Nepn*), which was recently identified by Mochida *et al*. as an inhibitor of Transforming Growth Factor-β signaling [52]. However, whether *5730521E12Rik *plays an inhibitory role in vivo during endoderm formation and patterning needs to be further investigated.

The new endoderm genes we identified in this study will provide a valuable tool for further investigation into the underlying molecular mechanisms that regulate endoderm development. In particular, the dynamic expression patterns of *Pyy*, *5730521E12Rik *and *Trh *from E6.5 to E9.5 provide intriguing insights into the endoderm fate mapping studies (Figure 4, 8 and unpublished data). In addition, *Cpn1 *and *1700011H14Rik *showed strikingly similar expression patterns suggesting they may be co-regulated. Further expression and functional analysis of many of these genes will give insights into endoderm development. Moreover, these endoderm genes could be valuable markers to assess and optimize ES cell *in vitro *differentiation into endoderm and endoderm derivatives.

## Conclusion

We identified novel as well as known genes that are expressed in DE progenitors by analyzing and validating DE longSAGE libraries. These genes provide a valuable tool for further investigation into the molecular mechanisms regulating endoderm development. Our study presents a successful application of analyzing and validating the large amount of data obtained by the Mouse Atlas of Gene Expression Project to identify tissue associated novel genes. The relatively high purity of the tissue source used for the construction of our DE longSAGE libraries and the comparison with a large number of longSAGE libraries from a variety of tissues and embryonic stages are the two critical factors for achieving an efficient screen.

## Methods

### Tissue collection and generation of SAGE libraries

Obtaining enriched DE tissue was achieved by a combination of proteolytic and manual micro-dissection methods [14]. E8.0–E8.5 embryos were isolated from timed pregnant female C57BL/6J mice. After removing the extra-embryonic membranes, the embryos were transferred to 1% trypsin/Hanks and incubated for 30 minutes on ice. Then 0.01%DNase/20% FBS/Hanks were added to block the activity of trypsin and to digest genomic DNA to reduce stickiness of the tissue. Next, using polished tungsten needles or fine tip forceps, endoderm and ectoderm were separated to minimize mesoderm contamination, and then transferred into Trizol (Invitrogen) (Figure 1A). Somite 0–6 endoderm pieces were pooled for the early whole endoderm library. Somite 8–12 endodermal portions were divided into foregut and hindgut region, and then pooled for foregut and hindgut libraries respectively (Figure 1B). In total, 3.5 μg, 2.7 μg and 3.5 μg total RNA were isolated from 124 early whole endoderm, 110 foregut and 115 hindgut pieces respectively. RNA quality was assessed using a Bioanalyzer (Agilent). Each SAGE library was constructed with 2.5 μg of DNA-free total RNA using the Invitrogen I-SAGE Long kit and protocol as previously described [32,33].

### SAGE data analysis

SAGE data was analyzed using DiscoverySpace software [53]. All SAGE libraries were generated by the Mouse Atlas of Gene Expression project [33]. They were filtered for sequence quality using a 95% quality cut off for all tags. Tag to gene mapping was performed using the mouse Refseq, MGC and Ensembl databases using the CMOST plugin in DiscoverySpace. Tags were considered sense position matches if they mapped in the same 5' to 3' orientation as the gene, and antisense matches if they mapped in the opposite orientation. Tag 'position' was determined by sequentially numbering NlaIII restriction sites from the 3'-most end (position 1) onward (i.e. next 5' tag would be position 2, and so on). A tag was considered unambiguous if it mapped to a single gene in a sense position and ambiguous if it mapped to multiple genes in a sense position.

### RT-PCR, whole mount in situ hybridization and histology

Semi-quantitative RT-PCR followed standard protocols. An ABI 7900 real-time PCR system (Applied Biosystems) and SYBR Green supermix (Applied Biosystems) were used for quantitative real time PCR. RNA from each tissue was prepared using Trizol (Invitrogen). Triplicate cDNAs were obtained by reverse transcription of 1 μg of total RNA from newly isolated batches of endoderm tissue. The primers used in the semi-quantitative RT-PCR and real-time PCR are listed in Additional file 3.

Whole mount *in situ *hybridization was performed as described previously [54]. Probe templates were generated by RT-PCR amplification from total RNAs isolated from E8.0–E8.5 endoderm, with average sizes of 400–800 bp, followed by sequence verification [see Additional file 4]. At least three embryos at each stage were examined for each probe, and restricted expression patterns were confirmed by independent sets of hybridizations. After whole mount *in situ *hybridization and photographing, the embryos were embedded by standard procedures in paraffin, sectioned at 8 μm, dried overnight, dewaxed in xylenes, and mounted for imaging.

## Abbreviations

SAGE: serial analysis of gene expression

DE: definitive endoderm

ES cell: embryonic stem cell

TFs: transcription factors

SPCs: signaling pathway components

## Authors' contributions

JH conducted most of the experiments and drafted the manuscript. AMC performed the SAGE data analysis. SCL carried out some whole mount *in situ *hybridization for the validations and proof-read the manuscript. MKW contributed to the design of dissection methods. YZ and MAM supervised the construction of the SAGE libraries. SJMJ supervised the creation of DiscoverySpace software for the SAGE data analysis. PAH conceived of this study, participated in the design of the project, finalized the manuscript and supervised the study. All authors read and approved the final manuscript.

## Supplementary Material

Additional file 1 Top foregut-specific and hindgut-specific tag sequences. This table shows the top 25 foregut specific and top 20 hindgut specific tag sequences with the counts and gene annotation. Click here for file

Additional file 2 Tag sequences enriched in the endoderm SAGE libraries. This table shows the tag sequences and annotations for transcription factors and signaling pathway components that are enriched in the endoderm with a T/L ration > 0.2. Click here for file

Additional file 3 Primers used for RT-qPCR. This file contains the primer sequences used for gene validation by quantitative RT-PCR. Click here for file

Additional file 4 Primers for amplification of the cDNA fragment for *in situ *probes. This file contains the primer sequences used to amplify DNA fragments used for *in situ *hybridization probes. Click here for file
