# Supplementary material for: A systematic screen for genes expressed in definitive endoderm by Serial Analysis of Gene Expression (SAGE)
Source: BMC Dev Biol. 2007 Aug 2;7:92. doi: 10.1186/1471-213X-7-92 (PMC1950885; doi:10.1186/1471-213X-7-92)
Supplement: Additional file 3 — Primers used for RT-qPCR. This file contains the primer sequences used for gene validation by quantitative RT-PCR. [file 1471-213X-7-92-S3.pdf]

Additional file 3: Primers used for RT-qPCR

gene symbol	primers	
	Forward	Reverse
Pyy	CAGTGGTGAAGACTCCCCCAAG	TGAACACACACAGCCCTCCAG
Trh	TGGTGCTGCTCTAGATTCCTGGAT	TTCGGCTTCAACGTCTTCCTCCTT
Prrx2	CCGTGCCTTTTCTCCATCACAG	GCCACCATAGCAGTGACTTGTTC
Otx2	GCTGAACATTCCAGTTTTAGCCAG	CTTTTTCCTTCTATGCCTCTCGG
Tbx1	AAGGCAGGCAGACGAATGTTC	GTCATCTACGGGCACAAAGTCC
Cyp26a1	CCATTCTTTGGGGAAACATTGC	TGCGTCTTGTAGATGAAGCCG
Hoxb6	GGTTCAATGGTAGATTCGCTGTCC	ATGTGCTCCTTCCAGTGGCTTTGG
Cdx1	GGAGAGTAGGCGGCATTGAAAG	AAGTGAGGCTGGAAGAGGAGACAG
