# Supplementary material for: A systematic screen for genes expressed in definitive endoderm by Serial Analysis of Gene Expression (SAGE)
Source: BMC Dev Biol. 2007 Aug 2;7:92. doi: 10.1186/1471-213X-7-92 (PMC1950885; doi:10.1186/1471-213X-7-92)
Supplement: Additional file 4 — Primers for amplification of the cDNA fragment for in situ probes. This file contains the primer sequences used to amplify DNA fragments used for in situ hybridization probes. [file 1471-213X-7-92-S4.pdf]

Additional file 4: Primers for amplification of the cDNA fragment for *in situ* probes

gene symbol	Primer	
	Forward	Reverse
Pyy	TCCTGCTCATCTTGCTTCGG	TGAACACACACAGCCCTCCAG
Trh	TGGATGGAGTCTGATGTCACCAAG	TGGCTCTTTGAAGTTCCTGAAGTG
Prrx2	CTACCACGATGAGCCCAGATTATC	GCCACCATAGCAGTGACTTGTTC
Otx2	GCTCAACTTCCTACTTTGGGGG	CTTTTTCCTTCTATGCCTCTCGG
Tbx1	AAGGCAGGCAGACGAATGTTC	TTCCGAGAGCGAGCAAAGGCACTC
Cyp26a1	GCAGGCACTAAAACAATCGTCAAC	ACATCGTGGGTGTCACAGATACTG
Hoxb6	AGGAAAAGCCAGCCGAGTGAAG	CAGCGAATCTACCATTGAACCG
Cdx1	CCGTCAAGGAGGAGTTTCTACCC	CGAGTTCTGTCTCAAGAGCAGTGG
Has2	CAAAGGAAAGTTGGAAAGCCACTG	CAAAGACTGCCACCATCTCTCC
5730521E12Rik	GCATTCCTTCCACCACCAAGAG	TGACAGGTAAAGATGGGACAGGTTC
Cldn9	ATACAGATGAGCGGGACCTAAGG	TGAACGGGAAGGGATGGAGTAG
Habp2	AAGACTGTATGTTTGCCCAGCG	CTTGTTGGTTCCCAGAGAGAAAAG
Spp2	GTGACAAGAATAAGACAGCCACCC	CAGTGAGCCCACACATCCTTTAC
Ttr	CCTCGCTGGACTGGTATTTGTG	TTGGGTTTTAGGAGCAGGGG
Cpn1	GGGTATCTGGTTGGTAGGAACAATG	GGGTCTTTCTTTCTGGATGTCCG
1700011H14Rik	AAAAGGCATCCACCCCGAAG	CAAGGCTATTCACCACAAGATTCC
Mogat2	CGCTCCTATCTGATGATGCTGAC	TGACTGCTTTTCCCCCATTCTAC
Spink3	GGAAGCACCCTGTATAGTTCT	GTTCAGATGCATTTATTCAAC
Phlda2	CATCCTCAAGGTGGACTGCG	CGGAATGGTGGGTTGGAAGC
Trap1a	CGCCCTTTATGAGGAGCAGTATG	AAAACAACCACTTCTTCCGCTG
Tdh	CCTGCGTTATCCTGGAATCATTTC	AGCATCGTAGCCACCAACTCTG
Lgals2	CCACCATTGTCTGTAACACCAGTG	CCTTGACCTTGGGGAAAAGC
Cubn	CCAGCAGGTCCAGATAACTGTGTG	TCGTTGCCCAAAAGAGTCCC
Pla2g12b	CTGTGATTCTCTGGCTGATACCG	AATGTGAGGTGTCAACTGGCAAC
Apoc2	GGCTTGATGAGAACAGGAGACTTC	TGATGCGAGCAAAAGAGGCG
Gabpb1	GGGAACTTCTCCACTTCATCTGG	TGTCAATACTTGCTGTCCATCGG
Arg1	GAAGAATGGAAGAGTCAGTGTGGTG	GAGGAGAAGGCGTTTGCTTAGC
Gm784	TTGCCGTTCACAGCCCTTAC	TTTGCTTGAGACAGTGCCCC
A230098A12Rik	TGGCGATTCTGATGGGGATG	TGGAGCAAAGGGAAAGGTGG
Usp22	TAGGCAGGGGATGGATGTCTTCAG	AACACAGAATGGAGGCAAGCGG
